# Theory In, Theory Out: The Uses of Social Theory in Machine Learning for Social Science

**DOI:** 10.3389/fdata.2020.00018

**Published:** 2020-05-19

**Authors:** Jason Radford, Kenneth Joseph

**Affiliations:** ^1^Department of Political Science, Northeastern University, Boston, MA, United States; ^2^Department of Computer Science and Engineering, University at Buffalo, Buffalo, NY, United States

**Keywords:** machine learning, computational social science, machine learning and social science, bias, fairness

## Abstract

Research at the intersection of machine learning and the social sciences has provided critical new insights into social behavior. At the same time, a variety of issues have been identified with the machine learning models used to analyze social data. These issues range from technical problems with the data used and features constructed, to problematic modeling assumptions, to limited interpretability, to the models' contributions to bias and inequality. Computational researchers have sought out technical solutions to these problems. The primary contribution of the present work is to argue that there is a limit to these technical solutions. At this limit, we must instead turn to social theory. We show how social theory can be used to answer basic methodological and interpretive questions that technical solutions cannot when building machine learning models, and when assessing, comparing, and using those models. In both cases, we draw on related existing critiques, provide examples of how social theory has already been used constructively in existing work, and discuss where other existing work may have benefited from the use of specific social theories. We believe this paper can act as a guide for computer and social scientists alike to navigate the substantive questions involved in applying the tools of machine learning to social data.

## 1. Introduction

Machine learning is increasingly being applied to vast quantities of social data generated from and about people (Lazer et al., 2009). Much of this work has been fruitful. For example, research using machine learning approaches on large social datasets has allowed us to provide accurate forecasts of state-level polls in U.S. elections (Beauchamp, 2017), study character development in novels (Bamman et al., 2014), and to better understand the structure and demographics of city neighborhoods (Cranshaw et al., 2012; Hipp et al., 2012). The increasing application of machine learning to social data has thus seen important success stories advancing our understanding of the social world.

At the same time, many (computational) social scientists have noted fundamental problems with a range of research that uses machine learning on social data (Lazer and Radford, 2017; Crawford et al., 2019; Jacobs and Wallach, 2019). For example, scholars have argued that machine learning models applied to social data often do not account for myriad biases that arise during the analysis pipeline that can undercut the validity of study claims (Olteanu et al., 2016). Attempts to identify criminality (Wu and Zhang, 2016) and sexuality (Wang and Kosinski, 2018) from people's faces and predicting recidivism using criminal justice records (Larson and Angwin, 2016) have led to critiques that current attempts to apply machine learning to social data represent a new form of physiognomy (Aguera y Arcas et al., 2017). Physiognomy was the attempt to explain human behavior through body types and was characterized by poor theory and sloppy measurement (Gould, 1996). It ultimately served to merely re-enforce the racial, gender, and class privileges of scientists and other elites. Today it is considered pseudoscience.

Acknowledging these misappropriations of machine learning on social data, researchers have sought out technical solutions to address them. For example, in response to claims that algorithms embedded in policy decisions often provide unfair advantages and disadvantages across social groups, some scholars in the Fairness, Accountability and Transparency (FAccT) community have proposed new algorithms to make decisions more fair. Similarly, researchers in natural language processing have proposed several new methods to “de-bias” word embeddings' representation of gender, race, and other social identities and statuses (Bolukbasi et al., 2016).

**Figure 1:**
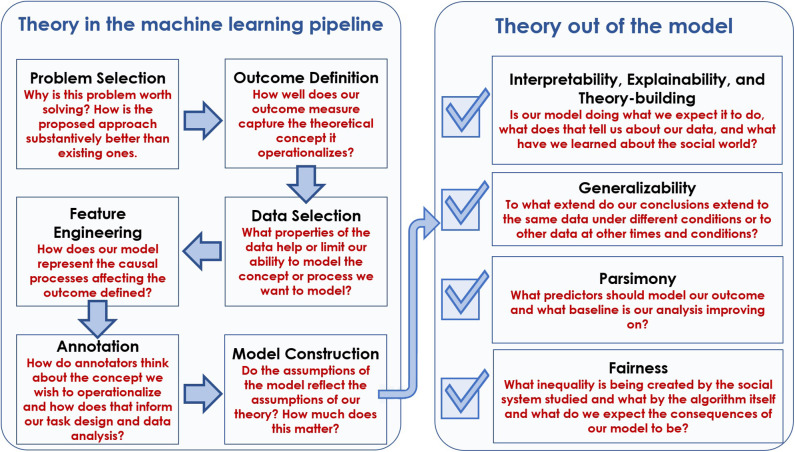
An overview of the Theory In and Theory Out sections of the paper. Each box within Theory In and Theory Out is a subsection. The box gives the name of the subsection and the claim we make about how social theory can be used to improve our technical solutions for addressing that problem. We see Theory In (model building) as a pipeline, and Theory Out (model use) as a checklist of things we might potentially want from the model and/or it's outputs.

The primary contribution of this paper is to put these challenges, criticisms, and searches for a solution into a single framework. Specifically, we argue and show that *at each step of the machine learning pipeline, problems arise which cannot be solved using a technical solution alone*. Instead, we explain how *social theory* helps us solve problems that arise throughout the process of building and evaluating machine learning models for social data. The steps in this process and an overview of how social theory can help us to perform the given step more effectively are outlined in Figure 1.

We define social theory broadly, as the set of scientifically-defined constructs like race, gender, social class, inequality, family, and institution, and their causes and consequences for one another. Using social theory in machine learning means engaging these constructs as they are defined and described scientifically and accounting for the established mechanisms and patterns of behavior engendered by these constructs. For example, Omi and Winant's (2014) *racial formation theory* argues that race is a social identity that is constantly being constructed by political, economic, and social forces. What makes someone “Black” or “White” in the United States and the opportunities and inequities associated with this distinction have changed dramatically throughout history and continues to change today. While there are other scientific definitions of race and active debates about its causes and consequences, engaging with them at each stage in the machine learning pipeline allows us to answer critical questions about what data we should use, features we should engineer, and what counts as fair (Benthall and Haynes, 2019; Hanna et al., 2019).

The paper is structured into two broad sections. In the *Theory In* section, we discuss how social theory can help us as we work through the model building pipeline. In the *Theory Out* section, we talk about a checklist of desiderata that we have for the models and results we produce, like generalizability, and discuss how social theory can help to improve these outputs of our work. Each subsection within Theory In and Theory Out focuses on a particular research problem or task and addresses a series of five questions:

1. What is the goal, or problem to be solved?
2. How have we tried to solve this problem computationally?
3. What are the limits to these technical solutions?
4. What solutions does social theory offer?
5. How can use social theories to solve these problems in our work?

In each subsection, we answer each question and use examples to illustrate our claims. Although each subsection addresses a unique element of Theory In or Theory Out, solutions identified in one step often enable us to address problems in others. For example, a model that is parsimonious is often more interpretable. A lack of a solution to a problem in one step can also prohibit a solution to issues that might arise downstream. At the highest level, a lack of social theory going into the model is likely to stymie drawing theory out. These overlaps are a strength, rather than a weakness, of the structure of this article. Like Olteanu et al. (2016), we believe that by emphasizing both the uniqueness and the critical relationships between different pieces of the pipeline, we can understand how the failure to address problems can propagate from one step to the next and ultimately impact what conclusions we draw from a study.

## 2. Related Work

Social scientists have long established that theory can solve methodological and analytic issues that new techniques cannot. For example, Small (2017) has argued that theory alone can address questions of how best to measure what it means for one to have a close social tie. In the present work, we regularly draw on this literature, seeing many parallels between prior methodological innovations like linear models and sequence analysis (Abbott, 1988, 1995).

Other scholars working at the intersection of machine learning and social science have also proposed important critiques which we draw upon throughout the paper. These critiques fall into four broad categories. First, scholars have argued that many machine learning papers focus too heavily on prediction relative to explanation (Wallach, 2018) or measurement (Jacobs and Wallach, 2019). The prioritization of prediction over explanation leads to models that perform well for unknown reasons, leading to *ad hoc* justifications for model decisions and performance. Prioritizing prediction over measurement leads to a failure to acknowledge the always imperfect association between what we are trying to measure and what we are able to quantify.

Others, like Nelson (2017), argue that machine learning applied to social data must come hand-in-hand with the development of new social theory. From this perspective, we do not necessarily know what model will work for what data, nor do we typically have theory to tell us what to expect. Consequently, we need to create new theory as we develop and run methods. This approach helps us to understand why machine learning models might present results that at first seem unintuitive, but that do reflect genuine patterns that should force us to reconsider our understanding of the social world. However, it also requires an a priori understanding of the potential theories that could apply, and seeks to adapt this existing theory, rather than create new theory entirely ex post-facto.

Still others have taken specific studies or sets of studies to task, arguing that they fail to understand the socio-technical context in which their data are produced (Lazer et al., 2009; Tufekci, 2014; Olteanu et al., 2016). For example, Tufekci (2014) argues that, despite generic claims above universal social behavior in many papers, research using Twitter data is unlikely to tell us much about social interaction on Facebook because the two have very different rules, data, and norms. Similarly, Olteanu et al. (2016) provide a dizzying array of potential pitfalls in analyzing social data, emphasizing the need for time-tested ideas from the social sciences, like matching and analyses of sample bias, to address these issues. These critiques point to the fact that machine learning is often done with one eye closed to the peculiarities of the data.

Finally, with the advent of algorithms that are making increasingly high-impact decisions, the FAccT community^1^ has arisen to study how these algorithms can serve to reflect social biases embedded in the complex sociotechnical systems within which they are embedded, and how we might be able to address these issues. However, recent critiques of the fairness literature argue that far too much emphasis has been placed on technical “solutions” to unfair and/or “biased” algorithms, relative to the structural causes and consequences of those algorithms (Green, 2018; Crawford et al., 2019; Hoffmann, 2019). Such scholarship has argued that social science disciplines need be at the forefront of our understanding of how to address these root causes.

Each of these critiques—that prediction does not equal understanding, that we must be ready to construct new theory to interpret our results, that myriad biases lurk in the data and methods we use, and that these methods can result in discriminatory outcomes with systemic causes—is critical in pushing us toward a better, more responsible application of machine learning toward social data. Further, the many works reviewed below that apply machine learning to social data with these issues in mind provide a further glimpse into the potential for this version of the science.

In the present work, we seek to unify these critiques, arguing that each of them are levied at different pieces of the same underlying problem—attempts to use technology, or *ad hoc*, post-ex facto reasoning, to address problems only social theory can solve. We argue below that theory alone can lead us to the valid explanatory models sought by Wallach (2018), to ensure we draw correct conclusions from initially unintuitive results, to help us characterize dangerous assumptions in our data collection processes, and to help us understand and address discriminatory, biased, or unfair model behavior.

## 3. Theory In

In this section, we discuss the pipeline for studies that use machine learning to analyze social data, from problem conception through model selection.

Throughout the section, two broad themes arise. First, given the breadth of data available to us, we sometimes act opportunistically; we use what we have to jump quickly on new problems. This push to rapidly solve pressing social problems by using widely available data and methods leads us to, for example, use a dataset to answer a research question that the dataset is ill-suited for. Problems can arise from these decisions that entirely undermine the utility of the research—for example, selecting a bad sample of data can undermine the external validity of a study.

Second, we often rely on intuition to make decisions about research design. For example, when constructing annotation tasks, intuition can lead to overly simplified designs, when many other potential approaches could also be equally, or more, valid (Joseph et al., 2017a). Often, these intuitions are good enough for the task at hand. However, when our intuitions are wrong, the results can be problematic. For example, following misguided intuitions about sexuality and its causes can lead to incorrect claims, made on top of poor research design decisions, about the biological nature of sexuality (Wang and Kosinski, 2018).

This combination of opportunism and intuition can be particularly pernicious when combined with a lack of theory. While social scientists also often rely on intuition (Tavory and Timmermans, 2014), they can rely on the scaffolding provided by previous theoretical work, guiding them toward a better research design and/or understanding of their data. In section 3, we discuss how we can use social theory to help us constrain our opportunism and intuitions to existing knowledge of the social world provided to us by social theory. This increases our chance at producing new, lasting science that helps move forward our understanding of society.

### 3.1. Problem Selection and Framing

As researchers, we are constantly asking ourselves, “what problem should we be studying?”^2^

Unfortunately, while technical approaches can sometimes help identify oddities in social data worth investigating, there is no technical solution to identifying good social science research questions. These insights require an understanding of what is known already about the social world, and where gaps in this knowledge lie. However, with the onslaught of big data, we all too often optimize for convenience, using the data we have on hand to study problems just because they seem solvable, and because they seem to have real-world relevance. For example, we use publicly available Twitter data to predict people's movements within cities (Bauer et al., 2012) or aggregated search data from Google Trends to predict the prevalence of the flu (Lazer et al., 2014).

This convenience approach to problem selection and framing leads to two problems. First, it can lead us to formulate and tackle problems that seem important but in reality serve chiefly as an exercise in prediction, providing little new insight into the social world. Second, it can lead us to address problems that our intuitions accurately assume are important, but leave us struggling to frame the reasons *why* the problem is important. Social theory can help to alleviate these issues.

First, theory tells us which problems are worth solving. For example, election prediction is an essential research tool because it provides a model for understanding political processes (Beauchamp, 2017; Kennedy et al., 2017). However, theory tells us that because of polarization, gerrymandering, and campaign finance laws, most American elections today are very predictable with only one piece of data—knowing who the incumbent is. Theory also tells us, however, that in nominally competitive races, *polling* provides the next best predictor, because politics is driven by opinion. However, polling is expensive, and s only available for the most high-profile races. Theory thus suggests that within the domain of elections, the correct problem to study is modeling opinion in competitive and under-polled elections.

Second, theory can help us to motivate and frame problems that seem intuitively important. It may be apparent that predicting the prevalence of the flu can help save lives. However, less obvious is what problem is being solved when predicting, for example, a person's political affiliation based on their social media behavior (e.g., based on their tweets) (Cohen and Ruths, 2013). However, recent work on political polarization urges us to study affiliation as a function of partisan identity (Levendusky, 2009), and shows that such identities are rapidly undermining social and cultural stability in the United States (Doherty, 2017). Social theory therefore explains why predicting political affiliation is important—in order to study its association with cultural polarization (DellaPosta et al., 2015).

Thus, while there may be situations in which the problem to be addressed can be motivated solely by the need for increased accuracy (e.g., correctly identifying a consenting individual's location information from WiFi signals), many machine learning problems can be made more interesting and relevant when grounded in underlying theory about the social behavior under study. There are many examples where scholars using machine learning on social data have used theory to identify and frame important problems. For example, several scholars have addressed precisely the problem of opinion polling in competitive and under-polled elections using big data (Wang et al., 2015; Beauchamp, 2017). And Cranshaw et al. (2012) take an intuitively interesting task—clustering venues on foursquare by the patrons they have in common—and ground it in theory from urban studies on the meaning of the term “neighborhood” to motivate and frame their work as addressing an unsolved problem of how neighborhood boundaries should be defined.

### 3.2. Outcome Definition

Having established a problem of interest, we turn to the task of defining and measuring our outcome. Our outcome measure is ideally meant to be the ground truth for our phenomenon we're modeling, i.e., an observation of the phenomenon itself. For example, if we are interested in studying partisanship, we can establish ground truth through a variety of means—whether someone votes for a single party (Poole and Rosenthal, 1991), who they donate money to (Bonica, 2014), or what topics they tweet about (Tsur et al., 2015). Unfortunately, this data is often not easily available. The easiest “technical solution” to this is simply to use a variable available in our data as ground truth, or, as we discuss in section 3.5, to construct the variable through a rapid crowdsourced annotation task.

However, this technical solution fails to help us fully characterize the link between the variable we select as an outcome and the concept we are interested in studying. For example, no technical solution can determine whether voting behavior or political sentiment in tweets is a more valid measure of partisanship (Cohen and Ruths, 2013). Answering these kinds of questions requires social theory. In this case, theory is needed to help identify what we mean by partisanship, or more specifically, by liberal vs. conservative. In turn, we must therefore approach ground truth as something being theorized by researchers. It therefore makes sense to do so in a way that existing social theory tells us is valid in capturing the construct we seek to measure (Hacking, 1986).

Returning to liberalism and conservatism, for example, political theories of partisan sorting and ideological alignment shows that people and sociotechnical systems shape the “ground truth.” Only recently have liberal and conservative labels for partisanship aligned with the Democratic and Republican parties in the United States—what is called partisan sorting (Mason, 2015). For example, Gentzkow et al. (2016) show that partisan ideology has only become distinguishable in Congressional floor speeches since 1980. That is, language has only become partisan in the past 40 years.

These theoretical insights, in turn, help us create a valid outcome. Instead of predicting liberalism/conservatism, a measure that has only recently come to align with partisanship, partisan identity theory (Van Bavel and Pereira, 2018) suggests we should instead focus on if someone is a Democrat or a Republican. Theory can further explain how to identify this outcome of interest in social media data. Specifically, partisan identity theory claims that party membership is driven by party identification. What makes someone a Democrat is not that they support public health care or market regulation but that they identify with Democrats. Thus, if we want to infer someone's political party identification from their tweets, we should look to whose side they take in a debate, rather than the specific issues they support. In his campaign, Donald Trump famously supported liberal policies like public health care and criticized the war in Iraq. These stances did not make him a moderate conservative. They made him a populist Republican as opposed to an establishment Republican.

### 3.3. Data Selection

The process of data selection is defined as the identification of one or more datasets that can be used to address the problem under study. Data selection is typically carried out using either precedent (i.e., using existing data) or convenience (i.e., using easily collectable data) as a heuristic.

This use of precedence and convenience stems from our interest not only in answering questions about the social world, but in desiring to do so via novel methodologies. For example, when constructing novel solutions to existing problems, we tend to reach for established datasets for which prior results exist for comparison. And for novel data collection, our methods often require large datasets, and so convenient collection of this data is almost a prerequisite.

But relying on either convenience or precedent can cause issues for social science questions, because all data contain both inclusions and exclusions that manifest in varying forms of bias (Olteanu et al., 2016). By taking shortcuts with data selection, we often choose to ignore or brush over these inclusions and exclusions. For example, Blodgett et al. (2016) show that language identification tools shown to perform well on a wide array of text corpora (Lui and Baldwin, 2012) suffer significantly at distinguishing African-American English as English in social media texts. Because scholars have often used this tool to filter out non-English tweets, the result is a set of studies on social media data where the voices of African Americans are diminished relative to white Americans.

As Blodgett et al. (2016) suggest, socio-linguistic theory could have helped us to anticipate the potential issues in using the convenient language classifier they studied to identify English vs. non-English content. Specifically, theoretical models of how dialects form emphasize that variations of written English may not readily align in terms of the primary features used by the language classification model, n-grams of characters (Rickford and Labov, 1999). Further, socio-linguistic theory emphasizing the importance of African American English and its distinctions from other English dialects in the presentation of the self online for Americans (Florini, 2014) would have emphasized the need for social media scholars to reconsider the notion that there is a single definition of English that they wish to study.

More broadly, then, social theory helps us to understand the implications of how we make our decisions on what data to include or not include in our sample. This is especially critical when we expect others will reuse our data or the models we construct from them. For example, a significant amount of research has used pre-trained word vectors from an unreleased Google news corpus, meaning the biases in the data are both unclear and unknown. On the contrary, Lundberg et al. (2019) use the statistical sampling and survey measurement theories baked into the Fragile Families Well-being Study to create the Fragile Families Challenge—a common data set computational social scientists can use to develop models predicting critical social factors like income, health, and housing. The use of theory to identify and explain important inclusion and exclusion variables have allowed research conducted during the challenge to contribute successfully to social scientific knowledge on child development (Salganik et al., 2019).

### 3.4. Feature Engineering

Feature engineering encompasses the process of converting our raw data to quantities that can be input into a machine learning model. The key question is, of course, how do we know that we have engineered the right features?

A technical solution to this question has typically privileged model performance. The problem with this approach to feature engineering is that the features we select might boost our performance but may not help us distinguish genuine from spurious signal. Overfitting to noise is one way in which injudicious feature selection can inflate performance. Another is to include features that are spuriously related with our outcome of interest or exclude features that are directly related.

Take the case of recidivism prediction as an example. To predict who will return to prison, not only do we need features that signal a person's propensity to commit a crime, but also features that capture police and judicial processes around who is likely to be arrested and convicted of a crime. For example, Sudnow's concept of “Normal Crimes” captures how the daily work of prosecution routinizes how certain kinds of cases from certain kinds of defendants are processed, in particularly who gets what plea agreements and whether jail time is recommended (Sudnow, 1965). Omitting features capturing both crime commission and criminal conviction yields a poorly-specified model that performs well.

Automated causal inference at scale is an as-yet unattained holy grail in machine learning. Thus, without theory, we cannot enumerate which features we should include and which we should exclude. Specifying the theoretical model in advance is the only way to enumerate what features we should generate (Rohrer, 2018; Pearl, 2019). Building theoretical models allow us to identify which features should be included, and if they are deemed important by a model, what they might mean. More concretely, Wallach (2018) argues that we should always be informing our selection of features and understanding of the problem with theory-based causal models in mind.

This argument is, of course, at odds with claims of “featureless” models, as many claim deep learning models to be. For example, where before we may have needed to provide a model for Named Entity Recognition with part-of-speech tags for each input word, modern deep learning architectures do not require this feature engineering step (Goldberg, 2016). However, even with such models, we are still making implicit decisions about our features, for example, by deciding whether to use words or characters as input to the model (Devlin et al., 2018). Further, the causal processes of interest often lay beyond decisions on whether or not to use words or characters. For example, regardless of what deep NLP model we choose to model an individual's language, word choices are often driven by more difficult-to-capture variables, like age and gender (Schwartz et al., 2013).

### 3.5. Annotation

Oftentimes we cannot identify some critical feature we want to model from our data. For example, Twitter does not provide data on the gender or religious affiliation of their users. In such cases, we often ask humans, be it ourselves or others, to go through the data and identify the feature of interest by hand.

When annotating data, a primary goal is to ensure that annotators agree. Due to both noise and intrinsic variation amongst individuals, different people look at the same data and come up with different labels. Our interest, particularly when searching for some objective ground truth, is to ensure that despite these differences, we can identify some annotated value on which most annotators roughly agree. Scholars in the social sciences have long established statistical measures of agreement in annotation (Krippendorff, 2004), which are readily used in the machine learning pipeline. However, machine learning researchers have also sought to increase agreement in various ways (Snow et al., 2008). These technical efforts to increase agreement largely rely on either trying to find the best annotators [i.e., those that tend to agree most frequently with others (Ipeirotis et al., 2010)], finding better aggregation schemes (Raykar et al., 2010; Passonneau and Carpenter, 2014), or simply by increasing the amount of data labeled (Snow et al., 2008).

At the core of many disagreements between annotators, however, is that the constructs we are seeking to annotate are often difficult to define. For example, Joseph et al. (2016) built a classifier to identify social identities in tweets, a concept that is notoriously varied in its meaning in the social sciences. Thus, even experts disagree on exactly what a social identity constitutes. Unsurprisingly, then, Joseph et al. found that non-expert annotators provided unreliable annotations, even after a discussion period. Annotations of hate speech have seen similar struggles, with limited agreement across annotators (Davidson et al., 2017) and with significant differences across annotators with different demographics (Waseem, 2016).

In such cases where the construct is difficult to define, technical solutions like adding more annotators or performing different aggregation schemes are unlikely to increase agreement. This is because, as with outcome definition, technical solutions cannot address the fundamental issue—defining the construct itself. In other words, technical solutions cannot be used to answer the questions, “what is a social identity?” Or, “what is hate speech?” Instead, we must rely on theory to provide a definition. For example, Affect Control Theory in sociology focuses not on the general idea of social identity, but rather on “cultural identity labels,” defined as “(1) the role-identities indicating positions in the social structure, (2) the social identities indicating membership in groups, and (3) the category memberships that come from identification with some characteristic, trait, or attribute” (Smith-Lovin, 2007, p. 110). Upon using this definition, and annotations from Affect Control theorists, Joseph et al. (2016) noted a significant increase in annotation quality.

Annotation, particularly with complex phenomena like identity, hate speech, or fake news (Grinberg et al., 2019), therefore requires starting with a theory of the construct we wish to measure and its intersection with the subjective processes of our annotators. One additional tool worth noting for this task that social scientists have developed is *cognitive interviewing* (Beatty and Willis, 2007). Cognitive interviewing involves talking to potential annotators about how they think of the construct, its potential labels, how they would identify those labels, and then having them actually try to apply our task to some test data. While similar to the idea of a pilot annotation task that machine learning researchers are likely familiar with, cognitive interviewing outlines specific ways in which theory can be applied before, during, and after the pilot to help shape the definition of the construct. Finally, although beyond the scope of the present work, it is also critical that annotation follows best methodological practices for structured content analysis in the social sciences (Geiger et al., 2019).

### 3.6. Model Construction

In building a machine learning model for social data, our goal is to predict, describe, and/or explain some social phenomenon. Our job, then, is to identify the model that best accomplishes this goal, under some definition of best. Our challenge is to determine which of the many modeling approaches (e.g., a deep neural network vs. a Random Forest) we can take, and which specific model(s) (e.g., which model architecture with what hyperparameters) within this broad array we will use for analysis.

It can be, and often is, overwhelming to select which model to use for a given analysis. Consider, for example, the goal of understanding the topics in a corpora of text. The early topic modeling work of Blei et al. (2003), has been cited over 28,000 times. Many of these citations are from extensions of the original model. For example, there are topic models for incorporating author characteristics (Rosen-Zvi et al., 2004), author characteristics and sentiment (Mukherjee, 2014), author community (Liu et al., 2009), that deal specifically with short text (Yan et al., 2013), that incorporate neural embeddings of words (Card et al., 2017), and that emphasize sparsity (Eisenstein et al., 2011). How do we construct a model that is best, or right, for our analysis?

O'Connor et al. (2011) describe this kind of modeling choice as occurring along two axes—computational complexity and domain assumptions. Computational complexity is used loosely to represent complexity in computational time and “horsepower.” Domain assumptions vary from few assumptions, essentially assuming “the model will learn everything,” to cases where we explicitly model theory. However, O'Connor et al. leave open the question of where in this space the “right” model for a particular problem is likely to fall, or how to define the right domain assumptions.

This is where theory comes in. By defining the goal of the model—prediction, explanation, description, and so on; and providing clear expectations for what our domain assumptions are, theory helps us navigate the computation/domain space. In the context of topic modeling, the Structural Topic Model (STM) (Roberts et al., 2013, 2014) provides a generic framework for defining our domain assumptions based on the factors we expect to be important for shaping the topics that appear in a document. By incorporating covariates into the modeling process that we theorize to be relevant, we can leverage theory both to create a model that “fits the data better,” and get outputs of the model that we can use to directly test extensions to our theory. The right model, then, is defined by theory. For example, Farrell (2016) uses theories of polarization through “contrarian campaigns” that originate in well-funded organizations to determine a particular instantiation of the Structural Topic Model that they use to study how polarization has emerged on the topic of climate change.

The STM is therefore useful in that, given an established set of generic modeling assumptions and a defined level of computational complexity, we can use theory to define the specific model we construct. Similar efforts have been made in other areas of text analysis as well. For example, Hovy and Fornaciari (2018) use the concept of homophily, that people with similar social statuses use similar language, to retrofit their word embedding model. This theory-driven change allowed the model to leverage new information, yielding a more performant model. As such, the use of theory to guide natural language processing models can serve as a blueprint for the application of theory in other domains of social data.

## 4. Theory Out

Machine learning has traditionally concerned itself with maximizing predictive performance. This means that the first results reported in machine learning papers, those in “Table 1,” are often a report on the model's predictive performance relative to some baselines. However, scholars are increasingly interested in other aspects of model output, like interpretability and fairness. In applied research, it is important for scholars to demonstrate that their model helps us understand the data and explains why particular predictions are made. These new demands for the output of machine learning models create problems for which technical solutions have been proposed. In this section, we argue that this technical innovation is insufficient on its own. We must engage with relevant social theories if we are to use our models to learn about social world.

### 4.1. Interpretability, Explainability, and Theory-Building

Few criticisms have been leveled against machine learning models more than the charge that they are uninterpretable. While a concrete definition of interpretability has been elusive (Lipton, 2016), the general critique has been that machine learning models are often“black boxes,” performing complex and unobservable procedures that produce outputs we are expected to trust and use. In trying to open the black box and account for our models, three distinct questions are often treated interchangeably:

- *What did the model learn, and how well did it learn it?* Meaning, given a particular input, how does the model translate this to an output and how accurately does this output match what we expect? We refer to this as the question of ***interpretability***.
- *Why did the model learn this?* What is it about the (social) world that led to the model learning these particular relationships between inputs and outputs? We will refer to this as the question of ***explainability***.
- *What did we learn about the world from this model?* What new knowledge about the social world can be gleaned from the results of our model? We refer to this as the question of ***theory-building***.

Interpretability, explainability, and theory-building get lumped together in the technical solutions that have been developed to open the black box. For example, sparsity-inducing mechanisms like regularization (Friedman et al., 2009) and attention (in neural networks; Vaswani et al., 2017) increase interpretability by minimizing the number of parameters to inspect. In turn, these technical solutions are used help us explain how the parameters relate to the data generating process (Zagoruyko and Komodakis, 2016). We also use model-based simulations, tweaking inputs to show how they produce different outputs (Ribeiro et al., 2016) and adversarial examples that fool the model to explore its performance (interpretability) the limits of its understanding about the world (theory-building) (Wallace et al., 2019).

However, while there are many methodological overlaps; interpretation, explanation, and theory-building are distinct research questions requiring different uses for social theories.

When interpreting models, social theory enables us to go beyond the technical question of *how* to look at the model to *what to look at*. In order to choose what parts of the model to visualize, we need to have pre-defined expectations about how the model is *supposed* to work and what it is *supposed* to do based on theories about how the phenomenon we're studying is represented in our data. For example, social theory, like Sen and Wasow's model of race as a “bundle of sticks” (Sen and Wasow, 2016), tells us that race is constituted by many different dimensions beyond just skin color. For example, racial bias driven by skin tone, called “colorism,” is different from racial bias driven by cultural codes like accent and hair style (Todorov et al., 2008). Consequently, if we want to understand how race is represented in a computer vision model, we should look to the different dimensions along which race is constructed. This can help differentiate, for example, whether biases in the model derive from cultural norms embedded in the population that make up the training data or from under-representation of individuals with certain skin tones in the training data (or both) (Benthall and Haynes, 2019; Hanna et al., 2019).

A good example of how theory can be used to guide interpretation is the work from Bamman et al. (2014), who identify tropes of literary characters. They validate their model by testing specific parameters against a slate of theory-based hypotheses. These hypotheses, derived from theory on the writing styles of authors during the time period of study, took the form of “character X is more similar to character Y than either X or Y is to a distractor character Z.” Good models were those that accurately predicted these theorized character similarities.

Oftentimes, we take our interpretation of model behavior and develop an account of why the model did what it did based on that interpretation. This often serves as an explanation of what the model did and an effort to build new theory. However, when we build explanations based only on model behavior, we are at risk of developing *folk theory* (d'Andrade, 1995). Folk theory involves leaning on common understanding to “read the tea leaves” (Chang et al., 2009) characterizing human behavior as simply “making sense.” This is dangerous, however (Kerr, 1998). Models will always output *something* and some model will always outperform others on some metrics. Building theory only from model output often serves to reinforce myths and biases.

For our explanations to contribute to a broader understanding of the social world, we need to not only find the right explanation for each model, but to also integrate many models and explanations into a coherent account of the world. Nelson's work on the development of second wave feminism is a prime example. She used social network and feminist theory to build different machine-learning based models for the structure of feminist communities in New York and Chicago. She then compared the structures of the social and idea networks to show that the ideas central to feminist community in New York were more aligned with what we understand today to be “second wave” feminism and that their community was more densely connected than that in Chicago. She argues this dense connectivity enabled feminists in New York to set the agenda for feminism in the 1960s and 70s.

Nelson and Bamman et al.'s work also provide a blueprint on how machine learning can help us to revise old or build new theory given empirical results from a machine learning model. To do so, their work tells us, one must first acknowledge the existing theoretical frames that have been used to characterize the problem. Following their work, one way to do so is to use these theories to generate hypotheses about what empirical results might look like, and to provide alternative hypotheses for what results might look like of a new or revised theory was instead true.

Another way to do so is to build a machine learning model that matches the theoretical model, and to then show that adding additional components to the model, inspired by new or revised theory, improve the performance of that model. For example, Joseph et al. (2017b) show that Affect Control Theory's (Heise, 2007) model of stereotyping may be insufficient by incorporating additional model components based on cognitive theories of stereotyping based on parallel constraint satisfaction models (Kunda and Thagard, 1996).

### 4.2. Generalizability

Generalizability refers to the goal of understanding how well results apply to the cases that were not tested. For example, if we develop a model to predict unemployment using mobile phone data in Europe (Toole et al., 2015), an analysis of generalizability might involve assessing whether the same approach would work in Algeria, Canada, or Mexico or on other kinds of data like internet searches or transit data.

In machine learning, generalizability is often addressed technically by reapplying the same methodology to other data to see whether it performs similarly to the original. For example, the generalizability of a topic model might be tested by applying a fitted model to different kinds of data. We also test the generalizability of a particular analytic approach by reapplying it in different domains. For example, Lucas et al. (2015) use machine translation across multiple languages to study whether politics in different countries were constituted by the same issues being discussed in the same ways. Finally, recent efforts have been made to train a model that learns representations of some universal input which can then be fine-tuned to apply to a variety of problems. For example, ResNet (Szegedy et al., 2017) and BERT (Devlin et al., 2018) learn generic representations for images and sentences, respectively, and can then be fine-tuned for various classification tasks.

While these technical solutions can make individual models more generalizable, they cannot help us establish why a result on one dataset can be generalized (or not) to others. For this, we need theories that tell us what similarities and differences are salient. Tufekci (2014) makes this point when arguing that we cannot treat one online platform (i.e., Twitter) as a stand-in for all others—as a *model organism* for society. The platform rules, social dynamics, and population that make Twitter worth engaging in for its users also distinguish it fundamentally from services like Facebook, Instagram, and WhatsApp. For example, theories of homophily suggests that, on any platform, people will associate with others like them. Yet, the commonalities on which we build connections depend on the platform itself. Our friends, colleagues, and public figures are on Twitter and our family is on Facebook. Following Goffman's theory of presentation of self, these differences in audiences drive people to behave differently on different platforms (Goffman, 1959).

Of course, there is no such thing as the perfect dataset, and science must be able to proceed in spite of this. Social theory can be used moving forward not as a way to find perfect data, but rather as a way to develop paradigms for understanding the particular strengths and weaknesses of different kinds of data, like data from different social media platforms, and for how models might be tweaked to generalize beyond the imperfect data they were trained on.

### 4.3. Parsimony

Parsimony refers to the goal of building a model with as few parameters as possible while still maximizing performance. Machine learning models benefit from parsimony because it decreases complexity and cost, limits the danger of overfitting, and makes it easier to visualize (Hastie et al., 2009).

A variety of technical approaches for constructing parsimonious models exist. For example, we can use regularization, topics, or factoring to reduce feature dimensionality. In the case of neural networks, we also use techniques like drop-out (Gal and Ghahramani, 2016) or batch normalization (Ioffe and Szegedy, 2015).

There are, however, three common flaws with these technical approaches. First, because many features are correlated with one another and the outcome, these approaches often arbitrarily select certain correlated features and not others. This arbitrary selection can make it difficult to differentiate between truly irrelevant features and those that are simply correlated with other relevant features. Second, decisions on when the model is “parsimonious enough” rely largely on heuristic comparisons between model performance on training and validation data [e.g., the “1-Standard Error rule” used in the popular glmnet package in R (Friedman et al., 2009)]. Finally, the standard machine learning assumption that we need many features can be incorrect even at relatively low values. It is often the case in social science problems that a small set of variables can easily explain a large part of the variance. A regularizer may select 1,000 features out of 10,000 while the best model may only need 50.

Social theory provides a solution to these issues by helping us define small sets, or “buckets,” of variables that we expect to explain a large portion of the variance in the outcome. Theories point us in the direction of the most important variables. Instead of starting with many features and trying to weed out irrelevant ones, we can use theory to create a baseline level of predictability from which we can assess whether additional features provide additional performance. Similarly, because theory provides us with the features we expect to be important, we may be able to identify cases in which regularization removes important, stable predictors due to correlation amongst variables.

The idea of identifying parsimonious, theoretically-informed baseline models for comparison has been shown to work well in practice. Theories of network centrality and homophily have proven to be robust predictors on a variety of tasks. For example, in their study of Twitter cascades, Goel et al. (2015) show that a simple model which accounts only for popularity of the user is an extremely strong baseline for predicting the size of a retweet cascade. These ideas align with theories of source credibility (Hovland and Weiss, 1951) and information spreading (Marsden and Friedkin, 1993).

Efforts to push the limits of predictability have informed the development of more formal social theory on the limits of predictability in social systems (Hofman et al., 2017), which may further extend our ability to estimate the degree of parsimony expected for particular problems. For example, in the Fragile Families challenge, the best submissions using thousands of variables and various models were not very good at predicting life outcomes like GPA, material hardship, and grit and were only marginally better than baseline models using only four variables (Salganik et al., 2020). In considering parsimony moving forward, we need to better understand the cases when the tools of machine learning add substantively to our model of the world beyond existing theory.

### 4.4. Fairness

In both popular media (Li, 2019) and academic literature (Mitchell et al., 2018), significant attention has turned to the question of how machine learning models may lead to increased discrimination against, or *unfairness* toward, certain social groups. The bulk of the work to ensure fairness has focused on making the input data more representative or modifying existing models to ensure fair outcomes (Kamishima et al., 2011; Kearns et al., 2017). Scholars have also recently focused on developing measures that account for sociologically relevant phenomena like intersectionality^3^ (Foulds and Pan, 2018), on the tradeoffs between existing measures (Kleinberg, 2018), and on a better understanding of the causal assumptions of different measures (Glymour and Herington, 2019) amongst other tasks.

However, as argued by a rash of recent work, there are important complications to defining fairness technically (Crawford, 2016; Barocas et al., 2017; Green, 2018; Selbst et al., 2018; Hoffmann, 2019; Mitchell et al., 2020). First, different people have different views on what is fair. Second, the views of those in power are the views that are most likely to be used. Third, models emerge from a vast and complex sociotechnical landscape where discrimination emerges from many other places beyond the models themselves. Finally, “Fairness” may not be the correct metric along which the harms of algorithms should be quantified. One conclusion has been that a fair algorithm cannot fix a discriminatory process. For example, recidivism prediction algorithms will almost certainly be used in a discriminatory fashion, regardless of whether or not the models themselves are fair (Green, 2018). We need social theory, e.g., critical race theory (Hanna et al., 2019), to better understand the social processes in which these algorithms and the data they are based on, are embedded. As this prior work has argued, social theory enables us to distinguish discrimination caused by the algorithm from that originating in the social system itself.

Perhaps equally important, theory can also help us to understand the *consequences* of unfair and/or biased algorithms. Take, for example, recent work showing that search algorithms return gender and race stereotypical images for various occupations (Kay et al., 2015). Social psychological theories, e.g., the Brilliance Hypothesis (Bian et al., 2017), focusing on representation emphasize that from a young age, we internalize representations of occupations and skills that cause us to shift toward those that are stereotypical of our own perceived gender. Thus, while technical solutions may help us to identify such problems, they cannot explain the impacts of these biases and thus why they should be addressed and how.

Finally, social theory helps to identify how unfair machine learning impacts our knowledge about the world. Biased algorithms, such as those that detect gender and race for demographic comparisons (Jung et al., 2017), can bias the science we produce. Standpoint theory and other critical epistemological theories have shown how who does science and whose data are used for what analysis affects what we know about the social world (Haraway, 1988; Harding, 2004). We do not want to replicate the patterns of exclusion and stigmatization found in the history of medicine (Martin, 1991), psychology (Foucault, 1990), and sociology (Zuberi and Bonilla-Silva, 2008) by throwing out data from marginalized people, only studying marginalized people as the Other, or not allowing marginalized people speak for themselves about their data.

Recently, similar critiques have been made by Jacobs and Wallach (2019). They argue that measurement theory, a particular domain of social theory engaging in the validity and reliability of different ways of measuring social constructs, can provide a concrete and useful language with which different definitions of fairness, and the impacts of algorithms, can be assessed. Their work provides an important example of how social theory can be used to bring old, socio-theoretic perspectives to bear in an area of current research in machine learning on social data.

## 5. Conclusion

The combination of machine learning methods and big social data offers us an exciting array of scientific possibilities. However, work in this area too often privileges machine learning models that perform well over models that are founded in a deeper understanding of the society under study. At best, this trade-off puts us in danger of advancing only computer science rather than both computer science and social science. At worst, these efforts push the use of machine learning for social data toward pseudoscience, where misappropriated algorithms are deployed to make discriminatory decisions and baseless social scientific claims are made.

However, as the many positive examples we have highlighted here show, machine learning and big social data can be used to produce important, ground-breaking research. To do so, the examples we highlight have baked social theory into each step of the machine learning pipeline. These works do not cherry-pick one theory, ex post-facto, to support their claims. Instead, they use multiple, potentially competing theories, at every step of the pipeline, to justify their inputs and help validate their outputs. In using, or at least acknowledging, competing theories, we can elucidate where disagreements exist and therefore which technical trade-offs are most important.

The positive examples we highlight, our review of negative examples, and the related work we draw on pave the way forward for the scientifically-grounded, ethical application of machine learning to social data. But our efforts must move beyond the way we produce research to the ways we review it, consume it, and encourage it as a research community. As reviewers, for example, we must ask ourselves if the work we are looking at is justified not only by statistical theory, but by social theory as well. And as a community, we must find ways to feature and promote papers that may not have the flashiest “Table 1,” but that provide a careful and well-grounded social scientific study.

Machine learning can and should become a critical piece of social science. The solution does not necessarily require a computer scientist to “go find a social scientist,” or vice versa. There is already a wealth of knowledge to draw from, and we should not allow ourselves or others to avoid delving into it simply because it is “out of our field.” For those who do not know where to start, we hope this paper is a guide to anyone for how to use that knowledge to address specific questions in the research. Similarly, social science should become an increasingly important part of machine learning. To be sure, certain problems faced by machine learning are computational issues (e.g., how to efficiently sample from a complex distribution) for which social theory will be of little use. But in incorporating social theory into their work, machine learning researchers need not reliquish model performance as the ultimate goal; we have argued here that, instead, theory can help guide the path to even better models and predictive performance.

## Author Contributions

All authors listed have made a substantial, direct and intellectual contribution to the work, and approved it for publication.

## Conflict of Interest

The authors declare that the research was conducted in the absence of any commercial or financial relationships that could be construed as a potential conflict of interest.

## Footnotes

^1^http://fatconference.org

^2^Importantly, not all researchers ask this question. Others, for example, may seek to understand the consequences of the practices they are studying. Thank you to Stef Shuster for pointing this out.

^3^Although see the critique from Hoffmann (2019).
